# Mapping urban living standards and economic activity in developing countries with energy data

**DOI:** 10.1371/journal.pone.0291824

**Published:** 2023-09-28

**Authors:** Felix S. K. Agyemang, Rashid Memon, Sean Fox

**Affiliations:** 1 Department of Planning and Environmental Management, University of Manchester, Manchester, United Kingdom; 2 Social and Economic Survey Research Institute, University of Qatar, Doha, Qatar; 3 School of Geographical Science, University of Bristol, Bristol, United Kingdom; National University of Sciences and Technology, PAKISTAN

## Abstract

Urban data deficits in developing countries impede evidence-based planning and policy. Could energy data be used to overcome this challenge by serving as a local proxy for living standards or economic activity in large urban areas? To answer this question, we examine the potential of georeferenced residential electricity meter data and night-time lights (NTL) data in the megacity of Karachi, Pakistan. First, we use nationally representative survey data to establish a strong association between electricity consumption and household living standards. Second, we compare gridded radiance values from NTL data with a unique dataset containing georeferenced median monthly electricity consumption values for over 2 million individual households in the city. Finally, we develop a model to explain intra-urban variation in radiance values using proxy measures of economic activity from Open Street Map. Overall, we find that NTL data are a poor proxy for living standards but do capture spatial variation in population density and economic activity. By contrast, electricity data are an excellent proxy for living standards and could be used more widely to inform policy and support poverty research in cities in low- and middle-income countries.

## I. Introduction

Rapid urban population growth in low and middle-income countries (LMICs) is contributing to the ‘urbanization of poverty’ [1]. Yet the true scale and nature of this challenge is unknown as we generally lack accurate and up-to-date data about household conditions in most cities in LMICs. Census data are infrequent and often undercount the urban poor [2–4], and traditional household surveys (e.g. Demographic and Health Surveys, or Living Standards Measurement Study data) generally do not have large enough samples to provide representative data for individual cities, let alone smaller areal units such as neighbourhoods. These traditional data sources are therefore of limited use in rapidly growing cities where policymakers need timely and spatially explicit information to support decision-making and efficient resource allocation. Given that virtually all projected population growth in coming decades will be absorbed by urban areas in low- and middle-income countries [5], urban data deficits are a significant obstacle to global poverty alleviation and development efforts [6].

Over the past decade, development economists have increasingly turned to non-traditional data sources, such as mobile phones, satellite imagery and volunteered geographic information. Of these, analysis of publicly available night-time light (NTL) emissions data captured by satellites has become the most popular means of estimating sub-national variation in economic activity and poverty in the absence of suitable administrative or survey data. It has long been recognised that energy consumption and economic development go hand-in-hand at the country level [7], and this simple insight inspired the use of NTL emissions as a proxy for economic activity in countries with limited data.

However, research to date has generally focused on the potential for NTL to capture production and income at regional scales. Yet there is also a well-established relationship between energy consumption and household welfare. Indeed, access to modern energy—i.e. energy from clean, affordable and renewable sources, such as electricity—is itself a key indicator of development [8–10], and was included as Goal 7 of the UN Sustainable Development Goals. Household energy consumption is closely associated with traditional measures of living standards, including income and consumption. While there is ongoing debate regarding the extent to which improved access to modern energy can drive improvements in household living standards [11–15], there is abundant evidence from experimental and observational studies that increases in income and assets are associated with increased total energy consumption (from all fuel types) at the household level. Importantly, rising living standards are associated with increased consumption of electricity in particular as households move up the ‘energy ladder’ (away from traditional biomass fuels) and diversify their ‘energy portfolios’ [16].

For example, a randomized controlled trial in rural India found that cash and asset transfers increased total household fuel consumption and specifically electricity for lighting [17]. Similarly, [18] used a Regression Discontinuity Design to measure the causal impact of a cash transfer program on fuel choice and expenditure in Pakistan and found a significant impact on monthly per capita fuel expenditure. These findings are consistent with observational studies based on survey data in wide range of urban contexts, including Brazil [19], China [20, 21], Ghana [22], India [23], Mexico [24] and South Africa [25]. The link between income and electricity consumption (as opposed to other fuels) is particularly strong in urban areas where access is near universal: globally, an estimated 97% of urban residents have access to electricity [10].

The strength of the income-energy consumption association varies by country and context (weather and socio-cultural factors are also significant) but is highly consistent within each context. Electricity consumption data may therefore be a useful proxy for household living standards in urban areas in the absence of detailed data on household income, assets or expenditure. It has the added advantage of being measurable in near real-time, which may be particularly useful for policymakers seeking information about the household-level impacts of economic shocks in LMICs.

There are two primary channels linking income to residential electricity consumption, both mediated by household appliances—particularly those associated with lighting, cooling, heating, washing, and refrigeration, which constitute a substantial proportion of total household electricity consumption. Rising income increases electricity consumption (a) directly due to greater use of existing appliances in the home (intensive margin), and (b) indirectly through the adoption and use of new appliances (extensive margin). Across countries there is roughly an S-shaped relationship between income and ownership of energy-consuming appliances, with low levels of ownership among at the bottom end of the income distribution, a sharp increase at context specific thresholds and a plateau in the upper deciles [26]. In low- and middle-income countries, the adoption and use of new appliances is a strong indicator of improvements in household living standards and can be observed indirectly with energy consumption data.

Could nightlights serve as a proxy for living standards as well as economic activity in cities in LMICs? Previous research has confirmed a strong correlation between NTL emissions and GDP at various spatial scales [27–30] and has used NTL emissions to produce subnational estimates of the incidence of poverty [31–33]. However, the accuracy of NTL predictions is highly dependent on both the context and scale of application [28, 34]. The extent to which such data could be used to identify variation in living standards or economic activity within cities in LMICs remains unclear [35]. We tackle the question in the context of Karachi, Pakistan using high resolution data from residential electricity meters. First, we demonstrate empirically that household electricity consumption is a strong proxy for living standards in Pakistani cities.

We find a modest association between NTL emissions and median monthly electricity consumption, but a visual inspection shows that even high-resolution NTL data disguise substantial spatial heterogeneity in living standards across the city and can produce highly misleading results. In contrast, proxies for population density, ‘establishment density’, social infrastructure, and road density explain over half of the observed spatial variance in NTL emissions. These results are consistent with those of Mellander et al (2015), which used highly detailed demographic and administrative data to test these associations in the Swedish context.

Our analysis makes two key contributions to the literature. First, we demonstrate that electricity data can be used to generate high-resolution areal estimates of living standards in urban areas in the absence of traditional sources such as census or administrative data. These small area estimates could be used to significantly improve the efficiency of social policy targeting in cities [36]. Second, we show that even relatively high-resolution night lights data (VIIRS) on their own are not suitable for estimating intraurban variation in living standards, even in very large cities. However, they do capture information on spatial variations in population density, infrastructure density and economic activity.

Georeferenced electricity data are not as readily available as NTL data and must be treated with care due to privacy concerns, but they do exist in all cities and should be available to the governments that either own or regulate supply networks. Given high rates of electricity access in urban centres, such data could serve as a valuable tool for policy makers and researchers concerned with understanding and raising urban living standards. We also show that volunteered geographic information, such as OSM data, may have useful modelling applications in cities with scarce data.

In the next section we present the methodology underpinning the paper. This is followed by results and discussion, structured into three subsections. The first subsection presents original analysis of nationally representative household survey data to empirically establish a close association between household electricity consumption and living standards in Pakistan and demonstrates the potential use of electricity data for mapping urban living standards. The second subsection compares NTL emissions and electricity consumption in Karachi using gridded data. The last subsection models NTL emissions at the cell level as a function of proxy measures for economic activity. Section IV concludes.

## II. Methodology for mapping living standards with energy data

We use high resolution data from residential electricity meters in Karachi, Pakistan to assess the potential of using NTL as proxy for living standard as well as economic activity. Karachi is a useful case study as it reflects many of the challenges facing urban planners and policy makers in LMICs: it is growing and changing fast, but data deficits obscure the nature of these changes. According to the 2017 census, Karachi has a population of 16 million, but this is widely believed to be an underestimate, and concerns about the accuracy of the census have led to a supreme court case [37]. Moreover, even basic population statistics and socioeconomic data have yet to be published as of December 2021. Nationally representative survey data are available every few years, but due to the sampling methodology these cannot be used to make inferences about household conditions within individual cities such as Karachi—only the urban sector in general.

To assess the correlation between NTL emissions (or ‘radiance’) and household living standards in Karachi, we exploit a unique georeferenced dataset containing information on median monthly electricity consumption from over 2 million residential electricity meters in the city. Previous studies have shown household energy consumption to be strongly correlated with traditional measures of living standards, such as income, consumption, and asset ownership—a relationship we confirm using nationally representative household data from Pakistan. Electricity consumption can therefore serve as a proxy for household living standards in the absence of spatially representative survey data within cities. We then develop a simple multivariate model to explain spatial variation in radiance drawing on OpenStreetMap data that reflect spatial variation in economic activity and infrastructure.

### Mapping living standards in Karachi, Pakistan with electricity data

The links between income, appliance ownership and electricity consumption noted above are clearly evident in Pakistan. To demonstrate this, we present original analysis of nationally representative survey data.

Our analysis uses data from the urban sub-samples of two rounds of the Household Income and Expenditure Survey (HIES) conducted by the Pakistan Bureau of Statistics. The HIES is conducted at regular intervals and generally has around 8000 observations for the urban areas. However, in 2015–16 an additional component, the Family Budget Survey, was added to the HIES to derive weights for rebasing price statistics. The urban sample was therefore roughly doubled to capture high price variation in urban areas. Our 2015/16 sample contains 15,934 observations while the 2018/19 sample contains 8805 observations.

First, to assess the relationship between appliance ownership and electricity consumption we construct a household Electrical Appliance Index (EAI) from the 2018/19 round of the HIES survey, weighting each appliance by the intensity of its energy requirements and expected daily usage. This was the only survey round for which the necessary asset data were available.

The 2018/19 HIES contains information on (a) appliances possessed in the last month and (b) appliances possessed in the last one year. Since the electricity expenditure data is available for the last one month, we use (a). Individually, washing machines, televisions and refrigerators are the most common appliances owned by households in the lowest deciles, with ownership of other appliances only increasing significantly after the sixth consumption decile. A similar pattern was found in India [38]. To construct the electrical appliance index, we weighted each appliance by the intensity of usage. Data on an appliances’ energy consumption were gleaned from several sources:

1. https://savejoules.com/refrigerator-brands.html This website provides a list of appliances manufactured by local brands, as well as their electricity consumption ratings. We chose the energy consumption of the median appliance to serve as weights.
2. https://energyusecalculator.com/electricity_microwave.htm This website was used for microwaves and irons since these were unavailable in the list in (1).
3. https://www.bijlibachao.com/air-conditioners/desert-air-coolers-better-option-than-air-conditioners-for-hot-and-dry-places.html This site was used to construct relative weights of air coolers.

The latter site was the most informal. However, air coolers are ubiquitous in lower middle-income classes in Pakistan and should not be ignored. But since these appliances are often made in small workshops rather than large factories, power ratings were difficult to come by. The final weights in estimated KWh per month are presented in Table 1.

**Table 1:** Electrical appliances and their expected power consumption.

Electrical Appliances	Electricity Usage (KWh per month)	Expected Usage (hours per day)
Television (CRT/ Flat Screen)	11	3
Fridge	73	24
Freezer	65	24
Washing Machine	31	1.5
Air Conditioner	127	8
Air cooler	21	8
Fan	22	10
Microwave	18	0.5
Iron	15	0.5

Next, we then calculated average monthly electricity consumption in kilowatt hours (kw/h) for each household from monthly expenditure on electricity using government notified tariff information from the respective rounds of the Economics Survey of Pakistan. Electricity consumption is measured at the household level to maintain consistency with the remainder of the paper which uses household level meter data.

The HIES surveys contain information on electricity expenditure in the month before the survey. We estimated units of electricity consumed by using the tariff structure announced by the Government of Pakistan, which took effect in 2019 (see Table 2). Since information on sales tax on electricity in 2015–16 was not unavailable, consumption was estimated using net expenditures in 2018–19 but gross expenditures in 2015–16. The HIES 2018–2019 began in July 2018 and ended in August 2019, which means the tariff structure changed in the middle of this survey round. However, it was possible to identify which households were surveyed in each year and we used the relevant price structure to estimate unit consumption. The distribution of the sample across different districts is available upon request. Table 3 shows the thresholds values for classifying households into electricity consumption deciles for each round of the survey.

**Table 2:** Tariff Structure announced by Government of Pakistan.

Tariff Slab	Tariff Effective June 2015 to December 2018 (Rs.)	Tariff Effective from January 2019 (Rs.)
Up to 50 units	2.00	2.00
0–100 units	5.79	5.79
101–200 units	8.11	8.11
201–300 units	10.2	10.2
301–700 units	16.00	17.6
Above 700 units	18.00	20.7

Source: Government of Pakistan 2019 and 2020 Consumer End Tariff Structure

**Table 3:** Threshold values for electricity consumption (Unit) deciles.

Unit decile	2015–16		2018–19	
	Min	Max	Min	Max
1	.85	73.80	18.22	87.45
2	74.98	102.37	87.59	132.66
3	102.90	123.45	133.70	153.47
4	123.98	155.07	154.51	184.69
5	155.91	176.15	186.45	212.65
6	176.67	197.22	213.06	229.19
7	197.54	231.31	229.44	270.56
8	232.15	273.21	271.39	311.71
9	273.88	363.05	312.88	402.81
10	365.40	3593.80	403.54	2987.95

Source: Authors’ calculations.

Importantly, electricity consumption varies by geography and season, and the HIES data were collected over the course of a year. This could introduce a significant source of measurement error given that respondents are asked only about the past months’ electricity expenditure. Despite efforts to account for this in the sampling procedure, we find some geographic bias in the season of enumeration. One district contains no winter observations and a further three contain no spring observations. More problematically, twenty percent of the sample was enumerated in the summer, when demand for electricity intensive cooling appliances is highest, and five of 29 districts were not enumerated in the summer at all. Since poor households’ electricity consumption would not change by much in summer and richer households’ electricity consumption would (due to a higher probability of owning air coolers or air conditioning units), this geographical imbalance may introduce a downward bias in estimates.

### Assessing the relationship between NTL and living standards within Karachi

To assess the potential for NTL data to serve as a proxy for living standards we compare high-resolution NTL data to georeferenced residential electricity consumption data in Karachi. There are two primary sources for NTL data used for economic analysis: the Defense Meteorological Satellite Program (DMSP) managed by the US National Oceanic and Atmospheric Administration (NOAA) and the Visible Infrared Radiometer Suite (VIIRS) instrument carried by a joint NOAA/NASA mission. Both products are publicly available. The DMSP data, which covered the period 1992–2013, have been more widely used by economists than the more recent VIIRS data, which begins in 2012 and runs to the present [39]. However, VIIRS data are increasingly preferred due to fewer errors and higher resolution, which is particularly important for subnational analysis and small are estimation (see [39, 40]). Given that we do not need a long time series but rather seek to validate the NTL data through cross-sectional analysis in 2019, we use the higher resolution VIIRS data. Moreover, it is unlikely that DMSP data could be used for intra-urban analysis of economic activity given problems with “blurring, coarse resolution, no calibration, low dynamic range, top-coding, and unrecorded variation in sensor amplification” [39]. By contrast, VIIRS data appear to do a much better job of capturing variation in economic activity within cities (ibid). For our statistical analysis we use average monthly radiance values from 2019 from the Annual VNL V2 product, which has a spatial resolution of 500m^2^ [41]. Median night light radiance values were calculated for each 500m^2^ cell.

Why might we expect NTL emissions to correlate with household living standards? In relatively densely populated areas, light is emitted from buildings, outdoor lighting infrastructure (e.g., streetlamps, signal towers etc) and vehicles. In theory, wealthier people may be likely to a) use more lighting, b) live in areas with higher quality lighting infrastructure, and c) use private vehicles. If true, it is possible that NTL emissions might capture variation in the relative wealth of neighbourhoods within a city. An obvious confounding factor is population density: in cities, poor people tend to live at higher densities than richer people (all else equal), and previous research has shown that NTL emissions capture information on population density [35, 40]. As a result, the amount of light observed in any given cell would likely be a joint function of relative wealth and population density. A second confounding factor may be the share of light being emitted from non-residential areas in a city. Factories, retail districts, and transport infrastructure are all examples of non-residential sources of light that may obscure any underlying association between residential energy consumption and NTL emissions. However, to date it has not been possible to directly assess the association between living standards and NTL emissions within cities in a LMIC context. Electricity consumption data allow us to make such an assessment.

The residential electricity data, which serves as our ‘ground-truthing’ layer, was provided by K-Electric (KE), the monopoly provider of electricity in Karachi. The data consist of average kilowatt hours (kw/h) of electricity consumed per month per meter between 1 April 2019 to 31 March 2020 (just before the economic impacts of Covid-19 became salient). The residential electricity tariffs are fixed across space at any given point in time in Pakistan, thereby eliminating potential biases associated with variation in price elasticities of demand across the city. Thus, whilst tariffs are not necessarily the same across Karachi, when prices change, all places in the city are equally affected. The raw data included 2.5 million residential meters in Karachi and surrounding rural areas. Observations falling outside of the urban boundary were discarded (313,446) leaving a sample of roughly 2.08 million. Of these, 136,088 negative values were treated as anomalies and discarded, along with all values between 0 and 1 (34,246). Values between 0 and 1 were dropped to eliminate plausibly unoccupied properties. A single unit of consumption translates into roughly 30 hours of use of a standard lightbulb in a month, which we take as a reasonable lower-bound threshold. The final sample consists of 2,048,418 observations. Finally, we calculated the median value of residential electricity consumption for each cell in our 500m^2^ grid.

We have a high degree of confidence that these data cover the overwhelming majority of households in Karachi. According to World Bank Development Indicators (accessed 8 March 2021) 100% of Pakistan’s urban population has access to electricity. Of the 8622 urban respondents to the 2018/19 HIES survey 98.11% reported having access to electricity from the grid. An independent survey conducted in one district in Karachi in 2018 (n≈1000) found that 96% of respondents received electricity bills each month [42, p. 85]. Importantly, the same study found that many people do not pay their bills regularly, resulting in bills that do not necessarily reflect actual consumption for any given time period. This makes billing data a less reliable source of information on electricity consumption than observed kilowatt hours consumed.

There are some notable limitations to this data, which are common in cities in many LMICs. While access coverage is generally comprehensive, illegal connections and sharing of connections (legal and illegal) are not uncommon and can lead to biased estimates. Meter data alone does not allow us to account for family size and composition, which can affect consumption patterns. Where relatively accurate population figures are available at small spatial scales, these unknowns could be partially accounted for, but this is rarely the case. Electricity consumption data are also likely to miss the most marginalised individuals and groups, who may have no access at all. Lastly, meters are not always located within the dwelling with which they are associated—there are meter junctions in some areas—limiting the extent to which a meter can be associated with a specific household or collection of households. Nevertheless, there are few other sources that provide the scale and spatial granularity of residential electricity consumption data, making it an attractive source for generating rough areal estimates of relative living standards in urban areas.

The data underpinning this paper can be accessed here: HIES; electricity consumption; NTL; and OSM.

## III. Results and discussion

### Electricity consumption and urban living standards in Pakistan

**Fig 1:**
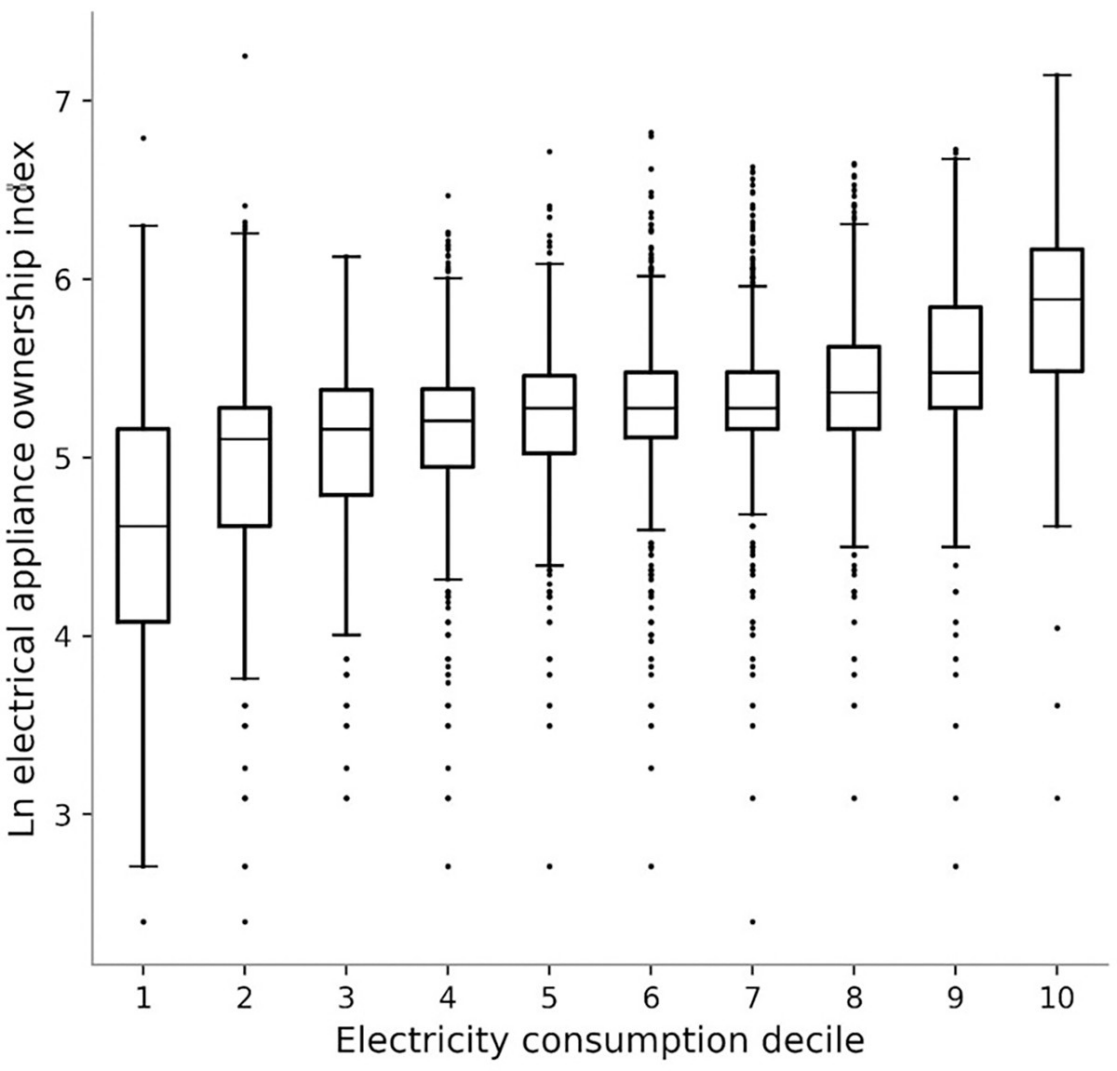
Household electricity consumption & electrical appliance index in 2018/19.

Fig 1 presents boxplots showing the association between household electricity consumption and the EAI, with households clustered into electricity consumption deciles. As expected, the association is positive (despite concerns about potential measurement error), with median values for the EAI increasing most abruptly in the lowest and highest deciles—consistent with previous findings. The Electricity Appliance Index explains 37 percent of the variation in household electricity consumption once family size and dwelling size are controlled for in a weighted OLS regression with clustered standard errors (results available on request).

Next, we construct an index of per capita household expenditure (on non-durables) as a proxy for living standards. Economists have long preferred a money metric of utility in the form of income or consumption [43], and consumption has overshadowed income as a measure of welfare at least since the World Bank started the Living Standards Measurement Surveys (LSMS) in the mid-1980s [44].

**Fig 2:**
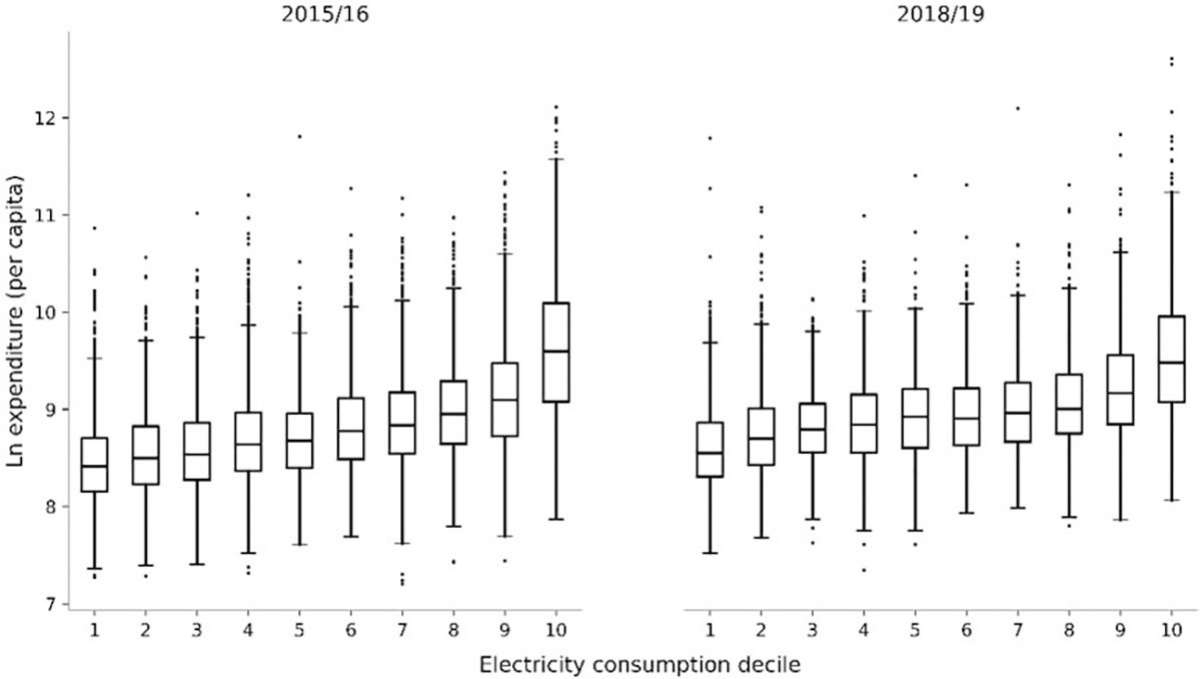
Household electricity consumption & expenditure per capita in urban Pakistan.

As with many countries, Pakistan uses the HIES to estimate consumption-expenditure based poverty lines. Our approach follows this tradition. We use the adult equivalency followed by the Government of Pakistan in setting its poverty line to compute family size: one child equals 0.8 adults. Here we can use both of the 2015/16 and 2018/19 samples to determine whether there is a consistent relationship between electricity consumption and household expenditure. Fig 2 presents boxplots showing the association between estimated household electricity consumption and per capita expenditure.

While not strictly monotonic (Median expenditure increases with electricity consumption decile in all cases apart from the 6^th^ decile of the 2018/19 survey round, which is slightly lower than the median value in the 5^th^ decile) the positive association between expenditure and electricity consumption is clear: higher levels of electricity consumption are systematically associated with higher levels of expenditure—our measure of living standards—across both the 2015/16 and 2018/19 survey rounds. Household level analysis shows that the correlation between per capita expenditure and electricity consumption was 0.5 in 2015/16 and 0.4 in 2018/19.

**Fig 3:**
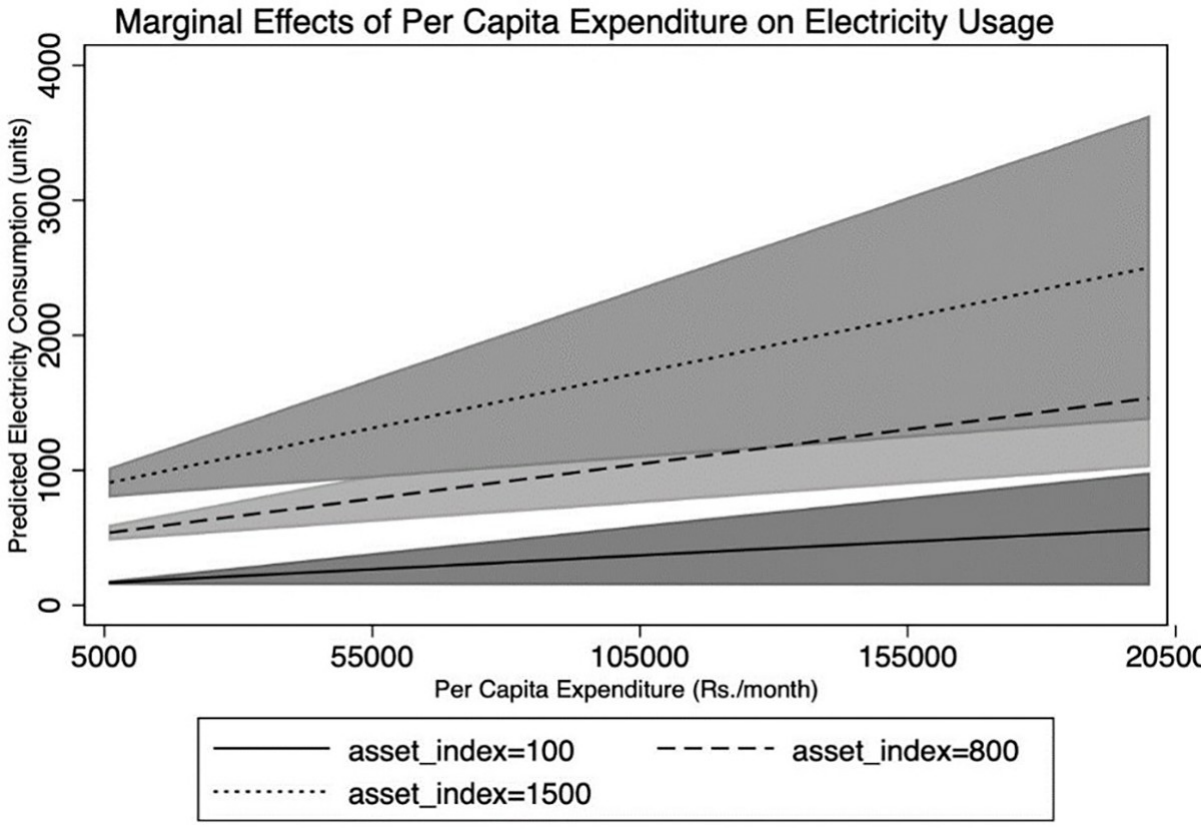
Marginal effects of per capita expenditure on electricity consumption.

Finally, we assess the marginal effects of per capita expenditure on electricity consumption holding the EAI constant at low, medium, and high levels. As shown in Fig 3, at any given level of electrical appliance ownership there is a positive association between expenditure and electricity consumption (i.e. positive intensive margin).

In sum, there is a strong association between electricity consumption, electrical appliance ownership and household expenditure. Given that household expenditure is a well-established and robust indicator of living standards, electricity consumption data can serve as a useful proxy indicator. However, measuring energy consumption with household surveys is costly, time-consuming and does not provide spatially explicit data at sufficient resolution to inform urban planning and policy making within individual settlements. Moreover, expenditure data from surveys can introduce measurement errors that can be avoided with direct observation of energy consumption. Direct observations of kilowatt hours consumed at the household level can therefore be used as a reasonably reliable proxy for living standards in the absence of detailed data on income and expenditure.

### NTL emissions and living standards

**Fig 4:**
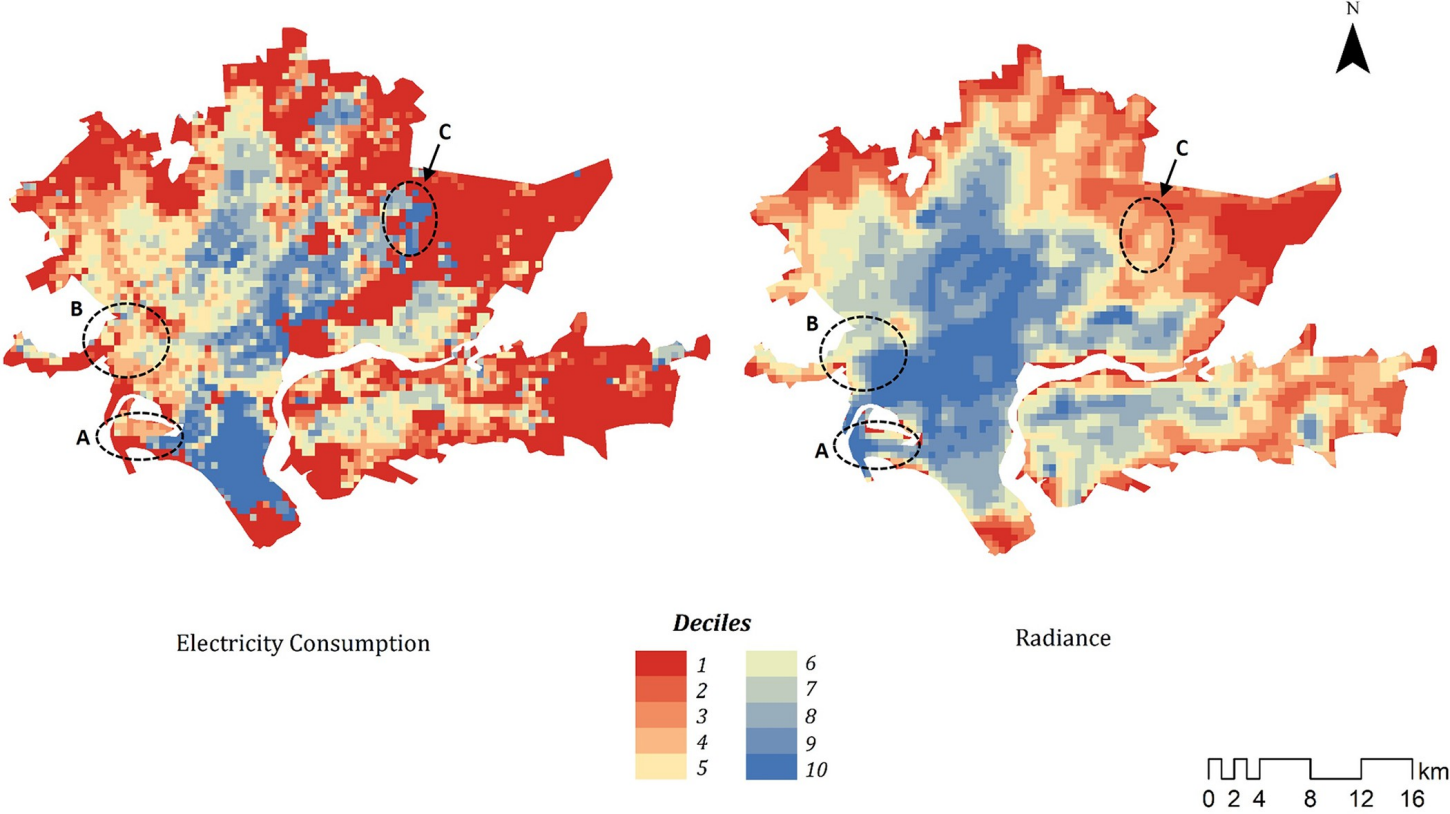
Spatial comparison of median electricity consumption and radiance in Karachi (deciles).

We calculated the median value of residential electricity consumption for each cell in Karachi (see section II). Table 4 shows that median cell radiance is positively and significantly associated with median residential electricity consumption (R^2^ = .19) in a simple bivariate regression. To assess the spatial pattern of this correlation, Fig 4 shows the radiance and electricity values mapped onto greater Karachi. Results are reported in deciles as we are primarily interested in relative living standards with clear thresholds—standard practice in living standards measurement for policy applications. This approach also strengthens privacy protections by expanding the range of potential values that could theoretically be attributed to any individual household within a cell.

**Table 4:** OLS model: Radiance & residential electricity consumption.

	*Dependent variable = Median electricity consumption*		
	(1)	(2)	(3)
Radiance	4.504*** (0.138)		4.601*** (0.164)
Meter count		0.059*** (0.004)	-0.005 (0.004)
*R* ^2^	0.19	0.05	0.19

*** p < 0.01

While there is broad correspondence in some central areas, there are also notable differences that illustrate the limitations of using VIIRS data as a proxy for living standards within cities. Area A shows a peninsula in Karachi South that contains diverse neighbourhoods. The south-eastern section contains a neighbourhood known as Clifton—one of the wealthiest in the city. On the north-west side are a collection of much poorer settlements adjacent to major port facilities. The electricity consumption data reflect these socioeconomic geographies while the radiance data would miss-classify Clifton as one of the poorest areas of the city and some of the poorer neighbourhoods as relatively wealthy. Area B encompasses the old city centre close to the commercial port, including areas such as Lyari, which is one of the most densely populated neighbourhoods in the city and contains a mix of poor and middle/upper-middle income residents. Again, the KE data reflect this diversity while the radiance data would rank this as one of the wealthier parts of the city. Finally, area C on the map contains Malir Cantonment—an area that contains both a military base and a civilian residential area that has some of the most expensive property prices in the city.

This visual comparison shows that while radiance is significantly correlated with electricity consumption (and by extension living standards) the spatial overlap is quite limited. As these case studies demonstrate, this appears to be caused by the fact that NTL emissions are capturing information about population density and non-residential sources of emissions. Small area population estimates from the 2017 Pakistan census are not available for Karachi and alternative products that provide such data from models (e.g., WorldPop) use NTL data as an input, creating concerns of endogeneity in this case. Given the near ubiquity of residential meters in the city, the meter count is the best possible proxy available.

We find that residential meter count per cell is more strongly correlated with radiance (R^2^ = .29) than median consumption values. This is consistent with case studies presented above. The wealthy neighbourhood of Clifton is less densely populated than those directly adjacent to the port; the neighbourhoods captured in area B are some of the most densely populated in the city; and Malir Cantonment (circle C) is effectively a low-density wealthy suburb.

If it is the case that wealthy people produce more light than poor people, all other things equal, then controlling for population density might yield a stronger association. In a simple OLS model with meter count and radiance as explanatory variables, and median consumption as the dependent variable, the R^2^ doesn’t change at all, and the meter count variable becomes insignificant (Table 4).

In sum, high-resolution NTL emissions do not appear to provide spatially accurate information about the distribution of living standards within a large city like Karachi. However, such data may still provide useful information on the spatial distribution of economic activity.

### Estimating NTL emissions with Open Street Map data

To assess the correlation between radiance and economic activity, [35] used high resolution data on the number of establishments, number of employees per cell, and wage bills (i.e. where wages were earned) as measures of activity in the Swedish context. Given that such detailed data are not available in Karachi, we extract georeferenced information on ‘points of interest’ (POIs) and land use from OpenStreetMap (OSM) to serve as proxies.

There are 105 types of POIs listed for Karachi, ranging from banks and supermarkets to schools and hospitals to fountains and parks. These were divided into three classes: Economic, Social Infrastructure and Other. We identified 60 Economic POI labels, including banks, ATMs, markets, retail shops, hotels, bakeries, etc. We classified 25 as Social Infrastructure, comprised of schools, medical facilities such as hospitals, government facilities, community facilities, and water and sanitation facilities. The 20 remaining miscellaneous POI labels were classed as Other and discarded from the analysis. In total, there are 9342 POIs in the cleaned dataset; 7145 of which are Economic, with the remaining 2197 falling into the Social Infrastructure category.

From these data we create measures of ‘local’ POI density and ‘neighbourhood’ POI density proximity on the assumption that the concentration of Economic and Social Infrastructure (ESI) POIs in a place is a reasonable proxy for economic activity. The ‘ESI POI count’ variable is the sum of all POIs *within* each cell; the ‘ESI POI count (buffer)’ variable captures all the POIs *outside* of the cell within a 708-meter radius of the edge of the cell. This ensures that all POIs in adjacent cells are accounted for. This neighbourhood measure is important because of overglow: light emitted in a cell may bleed into a neighbouring cell and increase the observed radiance value in that second cell. We expect a positive association between both the ESI POI count and radiance, as well as ESI POI count (buffer) and radiance. However, as the descriptive statistics in Table 5 show, the overwhelming majority of cells contain 1 POI or less. In the OLS model presented below we therefore include a simple dummy variable that takes a value of 1 if there are two or more POIs in a cell (‘ESI POI count (dummy)’).

**Table 5:** Descriptive statistics (*n* = 4588).

	Min	Max	Mean	Std	1^st^ Quartile	Median	3^rd^ Quartile
NTL average radiance	0	129	26	21	8	19	39
Median consumption (kw/h)	0	7013	155	221	0	133	224
KE meter count (population)	0	8794	487	839	0	39	732
Road density	0	.07	.02	.02	.01	.02	.03
ESI POI count	0	144	2	7	0	0	1
ESI POI count (buffer)	0	425	25	51	0	4	24
Distance to ESI POI	0	5562	617	834	0	279	937
Distance to economic land use	0	9248	1638	1599	404	1251	2438

We also calculate the Euclidean distance (in meters) from the centroid of each cell to the centroid of the nearest cell containing one or more ESI POIs to capture wider neighbourhood effects as the degree of overglow varies according to the brightness of lights being emitted. Finally, as a complementary measure of economic activity in the neighbourhood, we extracted data on the presence of commercial, retail and industrial land uses to identify cells that contain one or more of these. Many cells have economic land use classifications but no POIs. For example, there are large areas in Landhi, an industrial municipality in the south-eastern part of the city containing a mix of commercial and residential properties, which have no POIs but significant manufacturing facilities. Adding land use allows us to capture these economic activities that would otherwise be missed with sole reliance on the POI data. As with the POI distance measure, we created a variable that captures the distance between the observed cell and the nearest cell with an economic land use of any kind.

Finally, we extracted information on the road network and created a Road Density variable, defined as the total length of road observed within each cell divided by the cell area. To control for population density, we use the count of electricity meters per cell from the KE dataset. Table 5 summarises the data used in our model.

It is important to note that OpenStreetMap data are crowd-sourced and cannot be compared directly to rigorously catalogued and curated administrative data. There are unknown biases in the likelihood of any particular point of interest, or commercial establishment, or road being included in the data. The data cannot be treated as representative. However, the very nature of this data generation process may ultimately yield useful information for the application at hand: the probability that information will be voluntarily added to the opensource data is likely affected by the economic value or interest of the places and features added to the map. If NTL emissions reflect economic activity, and the density of information in any given place in the OSM database is at least a rough reflection of the density human economic activity in a place, we would expect to find a correlation.

Table 6 shows simple bivariate correlations between each of our explanatory variables and median cell radiance. The indicators with the greatest explanatory power are ESI POI count (buffer) (R^2^ = .39), KE meter count—our proxy for population density—(R^2^ = .29), ESI POI count (dummy) (R^2^ = .27), and Distance to ESI POI (R^2^ = .27).

**Table 6:** Bivariate correlations between radiance and economic proxy indicators.

	*Dependent variable = median radiance*		
	Coefficient	Std error	R-squared
KE meter count (population)	0.014***	0.000	0.29
Road density	749.158***	25.622	0.16
ESI POI count	1.170***	0.042	0.15
ESI POI count (dummy)	27.480***	0.665	0.27
ESI POI count (buffer)	0.259***	0.005	0.39
Distance to ESI POI	-0.0133 ***	0.000	0.27
Distance to economic land use (m)	-0.006***	0.000	0.19

*** indicates p < 0.01

Next, we create a multivariate model with two components: a ‘local’ component that includes the variables observed within the cells and a ‘neighbourhood’ component including the buffer and ‘distance to’ measures. As Table 7 shows, the local component accounts for just over 40 percent of variance individually while the neighbourhood component explains 54 percent of variance. When combined, the model explains just over 60 percent of variation in radiance. In an OSM-only model (i.e., omitting the proprietary KE data on meter counts) the model explains 58 percent of variation in observed radiance. All coefficients are significant and show the expected sign.

**Table 7:** Multivariate model of cell radiance.

	*Dependent variable = median cell radiance*			
	Local	Neighbourhood	Full	Full (OSM only)
Meter count	0.009*** (0.000)		0.006*** (0.000)	
Road density	174.392*** (26.066)		141.641*** (21.860)	331.359*** (19.900)
ESI POI dummy	18.823*** (0.654)		5.293*** (0.627)	6.743*** (0.644)
ESI POI count (buffer)		0.195*** (0.005)	0.144*** (0.005)	0.158*** (0.005)
Distance to ESI POI		-0.007*** (0.000)	-0.005*** (0.000)	-0.005*** (0.000)
Distance to economic land use		-0.003*** (0.000)	-0.003*** (0.000)	-0.003*** (0.000)
R^2^	0.41	0.54	0.61	0.58

*Note*: Standard error in parentheses.

*** p < 0.01

** p < 0.05

These results suggest that radiance is a joint function of population density, infrastructure density, and economic activity, as measured by the density of ‘points of interest’ and land use in and around cells. Our findings echo those of [35] who conclude that “light is a restricted proxy for economic activity at a micro-level” (pg. 5). The strong explanatory power of the ‘neighbourhood’ variables in this model also indicates high levels of spatial dependence in the radiance data, undermining confidence in cell-level inference at the scale of 500m^2^. Given this observation and the impossibility of untangling the demographic, infrastructural and economic sources of light, NTL data cannot be used in isolation to infer spatial patterns of productivity or living standards within large cities. However, it is likely that NTL data could be used as an input to multivariate models or machine learning models that use multiple inputs for estimation of economic variables.

## IV. Conclusions

Economic output and living standards at the household level are both closely associated with energy consumption. While night-time lights are a useful proxy for energy consumption, they are not suitable for estimating spatial variation in economic output or living standards at the intra-urban scale, even in a megacity such as Karachi. Instead, they reflect a combination of population density, infrastructure density and economic activity.

By contrast, we have shown that residential electricity consumption data are an excellent proxy for living standards, and there are clear benefits to using it. Electricity data can be used at a high spatial resolution and can theoretically be collected in real time. These are valuable attributes in rapidly changing cities in LMICs. Time-series consumption data could be used to address important empirical questions related to urban poverty, inequality, and urban economic development, as well as provide planners and policy makers with spatially explicit information about living standards. More precise point estimates could be used for specific research or policy applications, such as monitoring changes in local welfare due to an economic shock. Indeed, access to the data used in this paper was made available to explore its potential for providing policymakers with information for spatial targeting within the context of the COVID crisis. However, given the potentially disclosive nature of this type of data, great care is required in the reporting of such analysis to ensure maximum privacy.

Even accounting for measurement error associated with electricity theft, meter sharing and alternative fuel use, there are few sources of data that can match the spatial coverage of electricity data at such minimal cost. In cities where georeferenced household meter data are not available, or where the proportion of illegal connections is very high, data from transformers (which typically service between ~50–150 households) could be used in conjunction with population estimates or ancillary data such as satellite imagery to generate similar small-area estimates of living standards. Future research could assess this potential in a range of similar urban contexts in other countries, but research partnerships with public and private providers of electricity is required. However, such partnerships could rapidly improve our ability to measure and monitor energy poverty and living standards in urban areas in LMICs.
